# Proof of Concept: Extended Reality-Assisted Resternotomy Planning for Complex Cardiac Surgery

**DOI:** 10.21470/1678-9741-2024-0395

**Published:** 2026-05-06

**Authors:** Shay Illouz, David Mishali, Yisrael Parmet, Yael Ag. Rejuan, Lior Sasson, Hagi Dekel, Hanita Shai, Racheli Sion-Sarid, Alona Raucher, David Yogev, Netanel Nagar, Oliana Vazhgovsky, Yishay Salem, Erica Pollak, Alain E. Serraf, Leonid Sternik, Shai Tejman-Yarden, Eitan Keizman*

**Affiliations:** 1 Engineering in Medicine Lab (EiM), Sheba Medical Center, Ramat Gan, Israel; 2 Faculty of Medicine, Tel Aviv University, Tel Aviv, Israel; 3 The Edmond and Lily Safra Children's Hospital, Sheba Medical Center, Ramat Gan, Israel; 4 Department of Industrial Engineering and Management, Ben Gurion University, Beer Sheva, Israel; 5 Cardiothoracic Surgery, Wolfson Medical Center, Tel Aviv University, Holon, Israel; 6 Pediatric Cardiology, The Sylvan Adams Children's Hospital, Wolfson Medical Center, Holon, Israel; 7 Pediatric Intensive Care Unit, The Sylvan Adams Children's Hospital, Wolfson Medical Center, Holon, Israel; 8 Department of Radiology, Wolfson Medical Center, Holon, Israel; 9 Department for Cardiovascular Surgery, The Lev Leviev Heart Center, Sheba Medical Center, Ramat Gan, Israel

**Keywords:** Bioengineering (Incl Physical Modeling), Cardiac Anatomy/Pathologic Anatomy, Congenital Heart Disease, CHD.

## Abstract

**Introduction:**

Median sternotomy can cause postoperative adhesions, raising bleeding and organ damage risks during resternotomies. Computed tomography angiography (CTA) and extended reality (XR) are increasingly used to enhance surgical planning and minimize these risks. This study aims to assess the benefits of integrating XR technology into resternotomy planning

**Methods:**

This multi-center study, conducted at the Sheba and Wolfson Medical Centers in Israel, evaluated the utility of three-dimensional imaging in surgical resternotomy planning in 24 cases. Pediatric and adult patients selected for resternotomy underwent routine CTA, and those with adequate image quality were used to generate virtual three-dimensional segmentation. The images were evaluated preoperatively.

**Results:**

The findings indicated no significant benefit of XR over CTA in terms of resternotomy anatomical data. However, the accuracy of the XR models varied with medical experience: senior physicians rated the XR as less accurate for adult patients than did residents, but the ratings were high in both groups for pediatric cases. The XR models improved the surgeons’ understanding of chest anatomy in pediatrics more than in adult patients, whereas for surgical decision-making, XR was seen as more beneficial in pediatric cases, particularly by senior surgeons. Overall, senior physicians reported that XR influenced their surgical decisions more, suggesting that the utility of XR varies with physician experience and patient age.

**Conclusion:**

XR technologies have shown considerable potential in enhancing visualization and contributing to determining surgical strategies. However, the extent of their influence in terms of reducing operative durations and minimizing intraoperative complications requires further investigation.

## INTRODUCTION

**Table:** 

Abbreviations, Acronyms & Symbols				
AsAorta	= Ascending aorta		MV	= Mitral valve
Ao	= Aorta		MVR	= Mitral valve replacement
AR	= Augmented reality		PA	= Pulmonary artery
AV	= Aortic valve		PAt	= Pulmonary atresia
AVR	= Aortic valve replacement		PAB	= Pulmonary artery banding
AVSD	= Atrioventricular septal defect		POC	= Proof of concept
CABG	= Coronary artery bypass grafting		PS	= Pulmonary stenosis
ccTGA	= Congenitally corrected transposition of the great arteries		PV	= Pulmonary valve
CoA	= Coarctation of the aorta		RA	= Right atrium
CPB	= Cardiopulmonary bypass		RCA	= Right coronary artery
CT	= Computed tomography		RHD	= Rheumatic heart disease
CTA	= Computed tomography angiography		RIMA	= Right internal mammary artery
3D	= Three-dimensional		RS	= Resternotomy
DCRV	= Double chamber right ventricle		RSVC	= Right superior vena cava
DICOM	= Digital imaging and communications in medicine		RV	= Right ventricle
DORV	= Double outlet right ventricle		SMC	= Sheba Medical Center
DSS	= Discrete subaortic stenosis		SVC	= Superior vena cava
HLHS	= Hypoplastic left heart syndrome		TAPVD	= Total anomalous pulmonary venous drainage
IAA	= Interrupted aortic arch		TAPVR	= Total anomalous pulmonary venous return
ICMP	= Idiopathic cardiomyopathy		TV	= Tricuspid valve
IE	= Infective endocarditis		TVR	= Tricuspid valve repair
IHD	= Ischemic heart disease		VR	= Virtual reality
IVS	= Intact ventricular septum		VSD	= Ventricular septal defect
LIMA	= Left internal mammary artery		XR	= Extended reality
LV	= Left ventricle		WMC	= Wolfson Medical Center
LVAD	= Left ventricular assist device			

The median sternotomy technique was described by Milton in 1897 and reintroduced by Julian in 1957^[1]^. Today, this approach remains safe and efficient and is considered to be the gold standard for surgical treatment of all congenital and acquired heart diseases. Through this approach, the surgeon can see the entire heart and control the whole operative field visually and tactically^[2]^. However, postoperative adhesions often form between the heart and mediastinal structures and the sternum, which increase the risk of complications in subsequent sternal re-entries and dissection^[3^,^4]^.

The number of patients undergoing repeat sternotomies or resternotomy (RS) is on the rise^[4^,^5]^. Cardiac reoperation involving RS can be technically challenging and can result in major injuries to blood vessels and chest cavity organs. Traditional RS techniques provide poor visualization that can lead to inadvertent bleeding and prolonged operation times of up to 15% of all reported cases^[6^,^7]^. Despite the decrease in morbidity rates from 22.2%, in 1992^[8]^, and 19%, in 1999^[4]^, to 15%, in 2020, in the United Kingdom^[7]^, RS is still considered a dangerous procedure. The main structures at risk during sternal re-entry are the right ventricle (RV), which is typically attached to the sternum, as well as the ascending aorta, the innominate vein, and coronary grafts in instances of previous coronary artery bypass grafting (CABG) surgery^[9^,^10]^. In the pediatric population undergoing cardiac surgery, RS is even more frequent^[11]^. This is mainly due to the need for staged palliation or replacement of outgrown or degenerative prosthetic valves and conduits^[12]^. In children, although the estimated risk of injury during RS ranges are from negligible to low (1.3% to 5%), the results can still cause significant damage and thus must be addressed^[11^-^13]^.

Thorough preoperative evaluations of the retrosternal relations and dimensions are thus crucial for safe re-entry of the chest. It has become increasingly common to use computed tomography (CT) imaging for RS planning since it allows a detailed assessment of retrosternal relations and constitutes the safest and most detailed imaging technology enabling strategy planning when redoing cardiac surgery^[14^,^15]^.

The use of extended reality (XR) three-dimensional (3D) modeling, virtual reality (VR) planning, and augmented reality (AR) visualization preoperatively and intraoperatively in the field of cardiovascular intervention have all been shown to decrease surgical time and risk^[16^-^18]^. Even though XR technology may be one of the most promising techniques for assessing the anatomy of the chest cavity when planning for a RS, it is still not widely utilized.

This study aims to evaluate the efficacy of integrating XR technology into preoperative planning for RS in adult and pediatric patients. It compares the anatomical accuracy of XR-based 3D models with traditional CT imaging among surgeons of varying experience levels. Additionally, the study examines the impact of XR on surgical decision-making and operating room setup and investigates whether XR enhances the understanding of chest anatomy and reduces intraoperative complications. By addressing these objectives, the research seeks to provide measurable insights into the benefits and limitations of XR technologies in improving surgical planning and outcomes for complex cardiac surgeries involving RS.

## METHODS

This prospective-descriptive feasibility proof of concept (POC) multi-center study was conducted on both adult and pediatric patients in the Cardiothoracic Surgery Departments at the Sheba Medical Center (SMC) in Ramat Gan, Israel, and the Wolfson Medical Center (WMC) in Holon, Israel. The Institutional Review Boards of both institutions approved this study (SMC 7615-20, WMC 0148-22).

### Study Population and Design

A total of 24 RS cases were examined at SMC and WMC. Patient selection was made by the surgeons involved (David Mishaly and Leonid Sternik in SMC and Hagi Dekel and Lior Sasson in WMC), who identified candidate patients for RS. All candidates underwent a computed tomography angiography (CTA) scan as part of their routine preoperative surgical evaluation. Patients with an inadequate CTA scan resolution were excluded from this study.

In each case, after the patient was identified prior to surgery, the 3D imaging laboratory in each institution was asked to review the imaging and prepare a 3D segmentation of the sternum and adjacent mediastinal organs and vessels. The surgeon for each case evaluated the 3D images prior to operating. The 3D images were evaluated solely for purposes of this study and were not part of the preoperative planning protocol, so that none of the surgical plans were altered as a result of the 3D evaluation. After surgery, all the surgeons filled in an online questionnaire on the usefulness of the visualization (Appendix 1) adapted from Wellens et al.^[19^,^20]^ that assessed the accuracy of the 3D images with respect to the patients' anatomy and the surgeons' attitudes towards using an XR platform in the future.

Ten physicians participated in this study, and none had prior experience with medical XR platforms. Of these 10 cardiothoracic surgeons, four were residents in training, and six were senior surgeons. Twenty-eight questionnaires were collected covering all cases, in four cases both the resident and the senior physician answered a questionnaire. A Fisher's exact test indicated that no correlation (*P* = 0.6483) was found between physician status (resident or senior surgeon) or patient type (adult or pediatric).

### Patient Data and Imaging

Patient data, including baseline characteristics, chest CTA scans, surgical information, and surgical outcomes were extracted from the SMC and WMC medical databases. CTA images were acquired using high-resolution helical 16-slice CT with contrast enhancement, covering only a specifically defined field of view, and a 13-sec step and shoot acquisition (128 by 128 matrix, view angle three degrees, H mode) by a 256-slice scanner (Brilliance iCT; Philips Healthcare, Cleveland, Ohio, United States of America).

### Extended Reality and Three-dimensional Segmentations

**Fig. 1:**
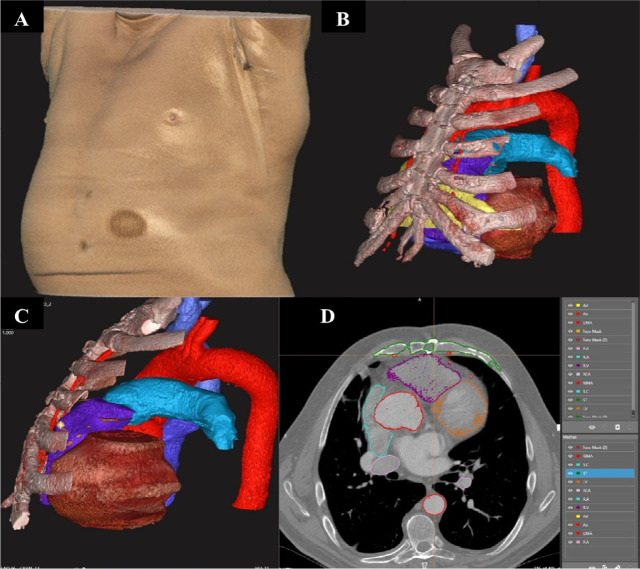
Screenshot of an adult resternotomy. A) An automated illustration of the patient's chest skin. B) The three-dimensional (3D) model in the same position as in 'A', includes the sternum, the adhesion (Ad) layer (in yellow), and the left and right mammary arteries (in red). C) The model rotated to the left, with white and yellow shading indicating the location of the right ventricle (RV) (in purple), positioned behind the sternum in the 3D model. D) Axial plane of the computed tomography segmentation and a window of the 3D meshes. Ao=aorta; LIMA=left internal mammary artery; PA=pulmonary artery; PV=pulmonary valve; RA=right atrium; RCA=right coronary artery; RIMA=right internal mammary artery; RV=right ventricle.

**Fig. 2:**
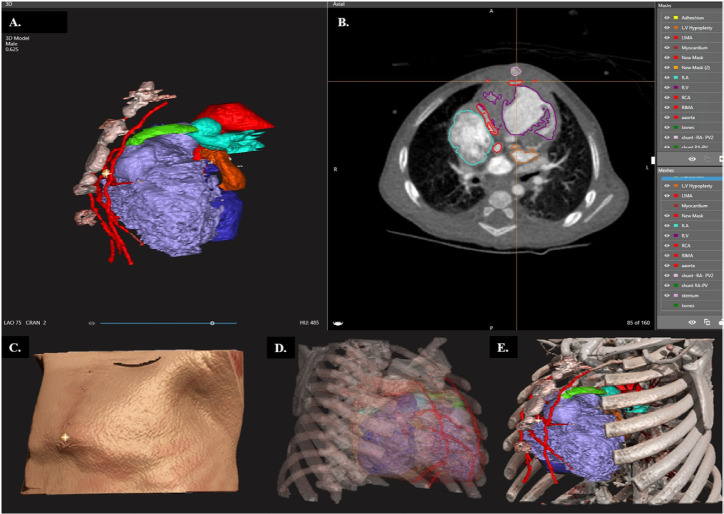
Hypoplastic left heart syndrome case study with multiple images from the D2P® slicer-software. A) White and yellow shading marking the coronary artery (in red) located behind the sternum on the three-dimensional (3D) model. There is a visible shunt (in green) connecting the right ventricle (RV) (light purple) to the pulmonary artery (cyan). B) Cross-sectional computed tomography image showing the same position as in 'A'. C) Automated illustration of the patient’s chest skin. D) 3D X-ray perspective of all the anatomical structures, viewed from left to right. E) The complete 3D model as presented to the surgeon. AsAorta=ascending aorta; LIMA=left internal mammary artery; LV=left ventricle; PV=pulmonary valve; RA=right atrium; RCA=right coronary artery; RIMA=right internal mammary artery; RV=right ventricle.

To create a 3D segmentation of the retrosternal anatomical structures, all the CT scans were exported without identifiers as digital imaging and communications in medicine (DICOM) files to dedicated computers at the SMC's Engineering in Medicine laboratory or the VR laboratory in WMC. Based on the latest CTA data, segmentation of the DICOM files was performed using D2P® software (3D Systems Inc., Littleton, Colorado, United States of America). The segmentation was meticulously conducted by a medical student, who identified and isolated key anatomical structures pertinent to RS, including the sternum, RV, adhesions, ascending aorta, and any existing coronary grafts from prior CABG surgeries. The segmentation process employed a combination of semi-automatic tools provided by D2P® for initial delineation, followed by manual refinements to enhance precision, particularly in areas with anatomical abnormalities or post-surgical alterations. To ensure the accuracy and consistency of the segmented models, each segmentation underwent a quality control process conducted by a senior cardiologist including the sternum and the retrosternal anatomical structures. Figure 1 presents the segmentation and 3D images of an adult patient prior to a CABG, and Figure 2 presents the segmentation and 3D images of a pediatric patient with a hypoplastic left heart syndrome, after a Norwood operation and Sano procedure being assessed prior to a Glenn operation.

**Fig. 3:**
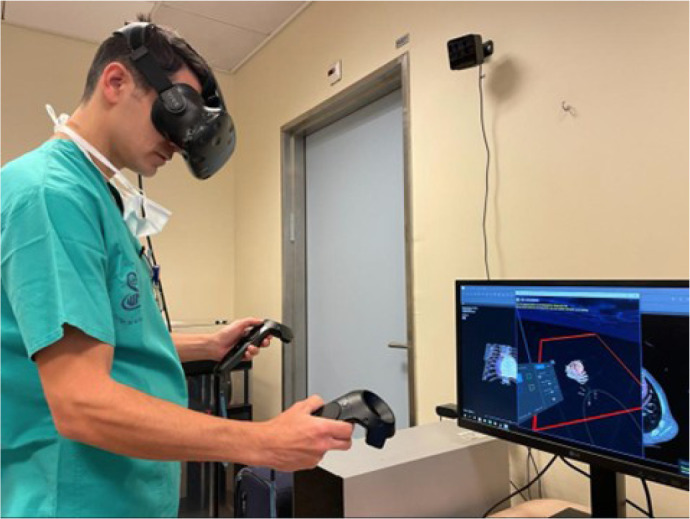
A resident surgeon training prior to resternotomy surgery.

To view the resulting segmentation, surgeons and residents in each institution used a dedicated VIVE system (HTC, San Francisco, California, United States of America) in a stereoscopic view using VR technology. Figure 3 shows a resident in cardiothoracic surgery examining a 3D model while wearing a VIVE headset.

### Sample Size Justification

This study is designed as a POC and feasibility investigation to explore the potential utility of XR technology in preoperative planning. Given its exploratory nature, a smaller sample size of 24 cases was deemed appropriate to gather preliminary data on the anatomical accuracy, impact on surgical decision-making, and overall utility of XR in both adult and pediatric populations. Additionally, involving 10 surgeons provided diverse perspectives and allowed for the assessment of XR's effectiveness across varying levels of surgical experience. Future studies with larger cohorts are planned to validate these initial findings and to perform more robust statistical analyses.

### Statistical Analysis

This POC study evaluated the value and efficacy of integrating new XR preoperational technology into cardiothoracic surgery. Ten surgeons completed a 25-item questionnaire on their experience with XR technology (Appendix 1). Generalized linear mixed models were used for the binary dependent variables (yes or no), and linear mixed models were employed for the continuous dependent variables. These methodologies were selected because they can address the case of repeated measures on a subset of physicians (multiple completion of the same questionnaire). The comprehensive model incorporated three fixed factors: physician identity, status, and patient type, along with their interactions. Variability across physicians was accounted for as a random factor. The analysis proceeded in two stages: the first involved testing for the significance of the random factor, which proved to be non-significant in all models, followed by assessing the fixed factors. The assessment began by calculating the significance of the interactions: if non-significant, the individual fixed factors were tested. Conversely, the significant interactions necessitated further post-hoc analyses of these interactions. All model fitting and follow-up analyses were done using R software version 4.2.2 and the libraries lme4, emmeans, and lmerTest.

## RESULTS

This multi-center study was conducted from July 2022 to December 2023. The patient cohort consisted of 24 cases, composed of five adults and 19 pediatric patients who were electively scheduled for cardiac surgery necessitating RS. Details of patient demographics, surgical procedures, previous interventions, and complications are listed in Table 1.

**Table 1:** The table below summarizes the details of patients who underwent RS as part of the study. The columns provide key information about each patient, including their age group, diagnosis, RS number, previous operations, current procedures, and any complications observed.

	Age^a^	Diagnosis^b^	RS^c^	Diagnosis	Previous operation^d^	Current procedure^e^	Complication^f^
1	Adults 1	PAt IVS	1	ICMP	CABG	LVAD	
2	Adults 2	IHD	1	IE	AVR	MVR	
3	Adults 3	TAPVD VSD	1	IHD	AVR CABG	CABG	
4	Adults 4	PAt IVS	1	RHD	MVR	MVR	
5	Adults 5	Tricuspid atresia IAA	1	Myxomatous MV disease	MV repair	MVR TVR	
6	Pediatric 1	HLHS	1	Subaortic stenosis	VSD	DSS and AV repair	Right atrial perforation
7	Pediatric 2	ICMP	1	Unbalanced AVSD DORV	PAB + CoA repair	Glenn	
8	Pediatric 3	IE	3	PAt IVS	Glenn	Fontan	
9	Pediatric 4	Subaortic stenosis	1	Perimembranous VSD partially closed by TV tissue, DCRV and DSS	VSD and DSS repair	DSS and VSD closure	
10	Pediatric 5	Unbalanced AVSD DORV TAPVD	1	Tricuspid atresia IAA	Norwood Sano	Glenn	Bleeding
11	Pediatric 6	DORV	1	HLHS	Norwood Sano	Glenn	
12	Pediatric 7	ccTGA VSD PS	1	HLHS	Norwood Sano	Glenn	
13	Pediatric 8	Unbalanced AVSD DORV	1	HLHS	Norwood Sano	Glenn	RV was severed but no bleeding
14	Pediatric 9	Tricuspid atresia IAA	1	Dysplastic aortic valve severe, postoperative regurgitation	Ross procedure	AVR	
15	Pediatric 10	HLHS	4	Unbalanced AVSD DORV TAPVD	AVR	Fontan Hraska	
16	Pediatric 11	HLHS	1	DORV	Glenn	Fontan	
17	Pediatric 12	IHD	3	Transitional AV canal	MVR	MVR	
18	Pediatric 13	RHD		Truncus arteriosus			
19	Pediatric 14	Transitional AV canal	2	DSS	DSS resection		
20	Pediatric 15	DSS		TAPVR			
21	Pediatric 16	TAPVR supracardiac type, verticle vein to innominate RSVC	2	TAPVR supracardiac type, verticle vein to innominate RSVC		Ostial resection of right and left pulmonary veins, sutureless technique and mobilization of atrial septum to the right	
22	Pediatric 17	Perimembranous VSD partially closed by TV tissue, DCRV and DSS	1	TAPVD VSD	PAB	PAt debanding Pulmonary artery banding	Bleeding during sternotomy
23	Pediatric 18	Myxomatous MV disease	4	Large VSD, severe tricuspid regurgitation, postoperative SVC syndrome, stuck TV	Redo TVR	Redo TVR	
24	Pediatric 19	Large VSD, severe tricuspid regurgitation, postoperative SVC, stuck tricuspid valve	3	ccTGA VSD PS	Glenn	Hemi-Mustard	

a Indicates whether the patient is an adult or a pediatric case;

b The primary condition diagnosed in the patient before the current procedure;

c Number of RS the patient has undergone, including the current one;

d Details of the most recent surgical procedure before the current RS;

e Surgical intervention planned for the current RS;

f Any complications encountered during or after the RS

AV=aortic valve; AVR=aortic valve replacement; AVSD=atrioventricular septal defect; CABG=coronary artery bypass grafting; ccTGA=congenitally corrected transposition of the great arteries; CoA=coarctation of the aorta; DCRV=double chamber right ventricle; DORV=double outlet right ventricle; DSS=discrete subaortic stenosis; HLHS=hypoplastic left heart syndrome; IAA=interrupted aortic arch; ICMP=idiopathic cardiomyopathy; IE=infective endocarditis; IHD=ischemic heart disease; IVS=intact ventricular septum; LVAD=left ventricular assist device; MV=mitral valve; MVR=mitral valve replacement; PAt=pulmonary atresia; PAB=pulmonary artery banding; PS=pulmonary stenosis; RHD=rheumatic heart disease; RS=resternotomy; RSVC=right superior vena cava; RV=right ventricle; SVC=superior vena cava; TAPVD=total anomalous pulmonary venous drainage; TAPVR=total anomalous pulmonary venous return; TV=tricuspid valve; TVR=tricuspid valve repair; VSD=ventricular septal defect

Ten physicians took part in this study; none had ever used a medical XR platform prior to this study. Of these 10 cardiothoracic surgeons, four were residents in training, and six were senior surgeons. Twenty-eight questionnaires were collected covering all cases, in four cases both the resident and the senior physician answered a questionnaire. A Fisher's exact test indicated that no correlation (*P* = 0.6483) was found between physician status (resident or senior surgeon) or patient type (adult or pediatric).

### Contribution of the 3D Model to Computed Tomography Imaging

This analysis examined the extent to which the 3D model provided significant additional insights beyond traditional CT scans. The results of the analysis of deviance table for the 3D model's contribution to CT imaging showed no significant effects of physician status (*P* = 0.4854) or patient type (*P* = 0.2136) on the perceived benefits. There was no significant interaction between physician status and patient type (*P* = 0.1868). This suggests that neither the experience level of the physicians nor the patients' demographics significantly affected the perceived additional value of the 3D model as compared to CT imaging.

### Contribution of the 3D Model to the Accuracy of the Anatomical Presentation of the Retrosternal and Cardiac Structures

There were significant differences in the assessment of the value of the 3D model in terms of accuracy as a function of physician status (resident or senior surgeon) and patient type (adult or pediatric). The linear model indicated a significant interaction between physician status and patient type (*P* = 0.02185) where senior surgeons rated the 3D model as less accurate for adult patients than the residents (mean estimate for a senior surgeon: 4.00 *vs.* residents: 8.50), with a significant contrast of 4.50 (*P* = 0.0067). However, for pediatric patients, the accuracy ratings for senior physicians and residents were similar (senior surgeons: 8.25 *vs.* residents: 8.60). Thus, senior surgeons considered the 3D model to be less accurate in adult cases but concurred with the residents for pediatric cases. Thus in adult patients where the anatomy is predicted and normal, the residents found the 3D useful for preoperative simulation while the senior surgeons found no added value to this novel modality; while in pediatric cases, where the anatomical valiance between the patients was significant, both the residents and the senior surgeons found the 3D useful as the anatomical structures adjacent to the sternum may vary substantially.

Moreover, the overall multiple R-squared value of the model was 0.3346, indicating that the model accounted for a moderate level of data variability. The F-statistics (4.022) with a *P*-value of 0.01883 also confirmed the model's reliability. Thus, overall, the perceived accuracy of the 3D model in identifying retrosternal cardiac structures appeared to be influenced by the physician's experience and the patient's age, with significant disparities particularly in adult patients between residents who found this modality helpful in all patients and senior surgeons who rated this modality accurate in pediatric patients.

### Contribution of the 3D Model to a Better Understanding of Chest Anatomical Orientation When Used as a Preoperative Simulation

The results showed that patient type significantly impacted how well the senior surgeons and the residents understood the chest anatomy they were examining (t = 2.504, *P* = 0.0189), as evidenced by the considerable difference in the means between adult (5.67) and pediatric patients (7.95). By contrast, physician status did not significantly affect the level of understanding (t = -1.370, *P* = 0.1876).

A type III analysis of variance further highlighted the significance of patient type (F = 7.0630, *P* = 0.01518), where again physician status and the interaction between physician status and patient type were not significant (*P* > 0.05). The model, when reduced to its simplest form, confirmed the influence of patient type on understanding the chest anatomical orientation when using 3D models. The significant difference between levels of understanding of adult and pediatric patients suggests that 3D models may be particularly beneficial in complex pediatric cases. 3D visualization provided the operator with a clear spatial understanding of the relationships between the atria, ventricles, and great vessels. It enabled assessment of the size and position of relevant structures requiring attention and even allowed simulation of the planned surgical procedure as described in the following section.

### Contribution of the 3D Model to Decisions as to the Surgical Approach

There was a significant interaction between physician status (resident or senior surgeon) and patient type (adults *vs.* pediatric) in evaluating the extent to which the 3D model could contribute to decision-making on the surgical approach. Senior surgeons found the 3D to be less useful for adult patients (estimated mean 1.00), but more useful for pediatric patients (estimated mean 8.33). The model's effectiveness was substantiated by an F-statistic of 16.91 (*P* < 0.0001), indicating a strong model fit. According to the estimated marginal means, residents considered XR to be a stronger factor in decision-making for adult patients (estimated mean 2.25). Contrasts between resident and senior physicians for pediatric patients (-4.03, *P* = 0.0001) highlighted the model's varying impact as a function of physician experience and patient age, thus underscoring its potential utility for residents performing pediatric surgery. This suggests that the 3D model's effectiveness in contributing to surgical decision-making was influenced by both the physician's experience and the patient's age.

### Changes in Surgical Decision Making as a Result of Extended Reality-Based Preoperative Planning

The results indicated a significant difference between resident and senior physicians in terms of how XR planning could potentially influence their surgical decisions. Specifically, senior physicians indicated that they would be more inclined to modify their surgical decision planning based on XR than the residents. This was evidenced by the notable shift in the logistic regression model's coefficient for senior physicians (estimate: 3.153, *P* = 0.00745), indicating a higher probability of decision-making changes. The difference in responses between resident (estimated in percents is 7.14%) and senior (estimated in percents 64.29%) physicians was statistically significant (z-ratio: -2.676, *P* = 0.0075), suggesting that experience plays a crucial role in how 3D models and XR technology is likely to influence surgical planning, regardless of patient age, rather the case complexity and anatomical variance.

### Changes in the Operating Room Setup as a Function of the Extended Reality Simulation

There was a clear divergence between resident and senior physicians: 76.29% of the senior physicians considered that the 3D model would have no impact on the setup, whereas only 6.808% of the residents believed it would not. This significant difference was supported by a logistic regression model showing a marked disparity in perceptions based on physician status (*P* < 0.0001). In terms of patient age, the influence of XR simulation was more pronounced in pediatric cases, with 59.09% considering there was a potential for change, compared to only 16.67% for adult cases. However, a Chi-square test indicated that this variance did not reach statistical significance (*P* = 0.07617), indicating that patient age was not a critical determinant in shaping perceptions of the influence of the XR technology on the operating room setup. It appeared that the setup for each patient was dependent on the surgeon's experience and readiness according to each surgeon's preference with varying changes according to the 3D simulation findings.

### Impact of Extended Reality Simulation on Changes in Decision-Making for Cardiopulmonary Bypass

Out of the 28 responses, 22 (78.57%) indicated that they did not consider that the XR simulation would lead to changes in cardiopulmonary bypass (CPB) decision-making whereas only six (21.43%) indicated they would consider changes. The fixed effects in the model (physician status and patient type) did not significantly predict potential changes in CPB decisions, as indicated by the high *P*-values and the boundary (-2.821) fit of the model. This indicates that whereas XR technology might be impactful in other areas of surgical planning, its influence on specific surgical decisions such as CPB usage was minor.

### Impact of Extended Reality Preoperative Planning on Shortening the Duration of Surgery in Resternotomy Cases

There was no significant influence of the interaction between physician status and patient type (*P* = 0.1462) on the potential to cut down the duration of RS. The fixed effects (physician status and patient type) had high *P*-values (physician status *P*=0.1795, patient type *P*=0.1835), suggesting that these factors were not statistically significant predictors of time reduction in RS procedures. The analysis of deviance of the model (Type II tests) reinforced these findings. The final step of the model, simplified to factor (X8) ~ 1, indicated a lack of significant predictors for the variables considered. This suggests that the potential time-saving benefits of VR technology in such surgical procedures may be independent of these specific factors.

### Differences in Avoiding Intraoperative Complications During Surgery

The analysis revealed that the interaction between physician status and patient type (Chi^^2^^ = 0.0000, *P* = 0.9995), physician status alone (Chi^^2^^ = 0.5964, *P* = 0.4400), and patient type alone (Chi^^2^^ = 0.0001, *P* = 0.9908) were not significant predictors of intraoperative complications. This outcome suggests that the presence of intraoperative complications was not significantly associated with the physician's status or the type of patient being treated. In adult cases, there were no reported complications. In pediatric cases, there were four complications: one case of right atrial perforation, another case where the RV was severed but no bleeding was recorded, and two cases of mild to moderate bleeding complications (Table 1). The right atrial perforation occurred despite the surgeon having predicted the risk of opening the chest before the surgery.

### Extent to Which Extended Reality Preoperative Planning Could Help Avoid Complications

The analysis indicated that neither physician status (t = -1.770, *P* = 0.0983) nor patient type (t = 0.235, *P* = 0.8161), nor their interaction (t = 1.947, *P* = 0.0685) significantly predicted the respondents’ assessment of a greater ability to avoid complications using XR. This was further supported by a Type III analysis of variance, which showed no significant effect for physician status (F = 1.2145, *P* = 0.31017) but a marginally significant effect for patient type (F = 4.8922, *P* = 0.04118) and an interaction between physician status and patient type (F = 3.7902, *P* = 0.06853).

### Surgical Confidence After Extended Reality Simulation During Surgery

The analysis revealed that in their assessments of confidence, the interaction between physician status and patient type was not significant (t = 0.029, *P* = 0.977). Additionally, neither physician status (t = 0.656, *P* = 0.521) nor patient type (t = -0.330, *P* = 0.744) significantly affected surgeon confidence. This suggests that the level of projected confidence in the use of XR as expressed by surgeons during these procedures may be influenced by other factors than physician status or patient demographics.

## DISCUSSION

This POC study examined the potential value of integrating XR technology as a complementary tool prior to RS in pediatric and adult patients.

Even though both the senior physicians and residents considered that the 3D images did not provide any new information beyond that obtained from the CT scan, the 3D images were considered to be more accurate, especially for pediatric patients. The fact the senior physicians felt that the anatomy was less accurate in adult patients might indicate a clearer understanding of VR's limitations in certain patient populations or a greater dependence on traditional imaging techniques by these more experienced practitioners. However, both residents and senior surgeons emphasized the accuracy of the 3D images for pediatric patients and acknowledged the importance of 3D configuration in understanding the chest cavity and the complex surgical issues associated with congenital defects. XR provides immersive 3D visualizations that allow surgeons to understand complex anatomical relationships better, which are often difficult to interpret using traditional 2D imaging. XR has wide applicability within structural and congenital heart diseases^[21^,^22]^. This improved spatial awareness facilitates precise surgical planning, potentially reducing operative times and minimizing intraoperative complications. Additionally, XR serves as an effective educational tool for residents, offering interactive learning experiences that deepen their understanding of intricate cardiac anatomies and surgical techniques^[21]^. Our pediatric cardiology department uses the same cases from preoperative sessions to teach residents and students during rounds, which is effective and does not require extra costs. This dual utility underscores XR's transformative potential in both surgical planning and medical education within cardiothoracic surgery.

More residents stated that they would have changed the surgical approach based on the XR simulation for both pediatric and adult patients, whereas the senior physicians tended to consider that XR would be instrumental in understanding and modifying the surgical approach for congenital RS patients, but not for adult patients. They also felt that XR-based preoperative planning could have had a greater impact on the overall surgical decisions, thus pointing to the differences in influence of new technologies as a function of medical experience. Even though both the senior physicians and residents thought that XR could impact the operating room setup, neither believed that incorporating this technology into the pre-surgical planning routine would alter the use of CPB, the duration of the operation, the potential for complications, or operator confidence.

A key finding of this study is the differing perceptions between senior physicians and residents regarding XR-based 3D models, especially in adult RS cases. Senior physicians rated XR models as less accurate for adult patients than residents. This discrepancy may arise from senior surgeons' extensive experience with traditional CT imaging and established surgical planning methods, making them more critical of new technologies like XR in less complex adult anatomies. Their reliance on proven techniques could lead to skepticism about XR's added value when conventional imaging provides sufficient detail. Conversely, residents' limited experience with RS nuances makes them appreciate innovative tools that enhance spatial understanding and surgical confidence. Zhang et al.^[21]^ (2023) reported that "sixty studies investigated surgical training whilst seven studies suggested that the use of XR-assisted technology increased surgeon confidence”.

XR technology emerged in our study as a valuable educational tool, which appeared to support surgical training and foster a more favorable perception among residents. Addressing these perception differences through tailored training programs that incorporate XR’s specific benefits for different patient types and surgical complexities, as perceived in this study, may promote broader acceptance and utilization of XR in adult and pediatric cardiothoracic surgeries. As noted by Tchervenkov et al.^[23]^ (2021), “There is currently a lack of internationally standardized criteria for training in congenital heart surgery around the world. There is a marked disparity between countries”. Rather than focusing solely on standardizing existing training methods for integrating technologies like XR, we propose this technology as an immersive solution to help bridge this gap. While our study did not directly evaluate training curricula, the immersive nature of XR-based preoperative planning may offer an opportunity to reduce these training disparities by providing consistent, anatomy-based clinical case-learning experiences across varying clinical environments.

Overall, however, both the senior physicians and the resident surgeons agreed on the value of XR technology in RS for congenital heart disease. This finding is consistent with expectations, given the increasing number of recent studies that support the integration of XR in congenital heart disease^[22^,^24]^. These observations also align with a recent review on procedural planning in structural heart disease, particularly in scenarios demanding a comprehensive understanding of complex anatomies^[22]^. XR's immersive and detailed 3D visualization offered critical insights, which were often less discernible in traditional imaging modalities. The findings showed that the surgeons' understanding was substantially enhanced by the XR's ability to display unattainable views in the operating room, such as the proximity to 'unseen' surrounding structures^[24]^. Thus, XR may be a significant aid in the planning of complex surgical cases, especially in pediatric contexts, where its potential benefits for both RS planning and cardiac anatomy analysis decision-making were considered to make it more accurate, safe, and efficient, thus substantially outweighing the associated cost^[25]^. During this study, the surgeons as a whole were eager to participate and enthusiastic about testing more cases. Most of the surgeons indicated a preference for having a XR platform available during their next RS and have been requesting access regularly since the study was conducted. As a result, in the two hospitals where XR was tested, this technology has been incorporated into the routine preoperative planning for RS. Nevertheless, despite the positive results, further research is needed to examine the added value of XR to the retrosternal modeling as well as to the whole cardiac and mediastinal anatomy. Long-term or randomized control studies could also be conducted to investigate the impact of XR and AR on surgical outcomes, which include the potential to decrease surgery time and complications and improve the general safety of the operation. Developing more automatic and quicker segmentation tools and investigating them in future research are likely to play a crucial role in the integration of this platform in daily practice.

### Limitations

This study has several limitations, including its small sample size of 10 surgeons who completed 28 questionnaires, which could affect the generalizability of the findings. Hence these preliminary results cannot be interpreted as indicating extensive acceptance of XR for these purposes. Furthermore, since this is relatively innovative technology, half of the surgeons had no prior experience with XR. Thus, the “wow” factor of fancy visualization technology might have influenced their perceptions and their assessments of the potential value of the platform^[26]^. The use of a questionnaire for data collection and the fact that some surgeons completed it several times could have introduced bias due to its subjective nature.

## CONCLUSION

The findings point to the potential value of XR technologies in preoperative planning for RS. XR technologies have shown considerable potential in enhancing visualization and contributing to determining surgical strategies. XR-based 3D models provided surgeons with a superior spatial understanding of complex anatomical structures compared to traditional CT imaging, leading to more informed surgical decision-making and optimized operating room setups. The enhanced visualization capabilities of XR were especially beneficial in pediatric cases, where anatomical variations are more pronounced. However, the extent of their influence on reducing operative duration and minimizing intraoperative complications requires further investigation. Focusing future research specifically on the intraoperative phase could unveil XR's capabilities to lessen complications.

## Data Availability

The authors declare that the data will only be available upon request to the authors.

## Artificial Intelligence Usage

The authors declare that no artificial intelligence tool was used in the preparation of this article.

## Appendix 1

Resternotomy Questionnaire:

1. What is your title? Senior surgeon/ Resident physician

Was the VR simulation used preoperatively or postoperatively? (pre/post)

**Senior Surgeons Only**

2. Do you have any experience with VR medical systems? (y/n)

3. If you have experience with VR, how many hours of VR use have you accumulated?

4. Was femoral cannulation performed before the sternal incision? (y/n)

5. If yes, did you ask the perfusionist to empty the heart before dissecting adhesions? (y/n)

6. To what extent did the VR model help you decide on the surgical approach?

7. Did the VR simulation change the operating room setup in any way? (1-10)

8. If yes, please elaborate:

9. Did the VR simulation change decisions on CPB use? (y/n)

10. If yes, please give specifics:

11. In your opinion, to what extent was the VR model accurate in terms of the location of the retrosternal cardiac structures? (1-10)

12. In your opinion, did the preoperative VR planning lead to a change in the decision? (y/n)

13. To what extent did the VR model add value to CT imaging? (1-10)

14. In your opinion, could the VR-based preoperative planning shorten the duration of the resternotomy? (y/n)

15. If so, how much (in minutes)?

16. Were there any intraoperative complications during the surgery? (y/n)

17. If yes, please specify:

18. In your opinion, could the VR-based preoperative planning help you to avoid complications? (1-10)

19. To what extent could the VR model give you a better understanding of the chest anatomical orientation if used as a preoperative simulation? (1-10)

20. Please rate your confidence in the VR simulation during the procedure. (1-10)

21. Would you say the VR could make you more confident during chest surgery if used as a preoperative simulation?

22. Mark the extent to which you would like to use this VR simulation before your next resternotomy. (1-10)

23. Mark the extent to which you would like to use this VR simulation prior to chest operations in general. (1-10)

24. To what extent do you feel VR is an effective tool for preoperative planning in chest surgeries? (1-10)

25. To what extent do you feel the VR simulation can improve residents' training on chest surgery? (1-10)

**Residents Only**

26. Was the VR simulation used preoperatively or postoperatively? (1/2)

27. What year of residency?

28. Do you have any previous experience with VR systems? (y/n)

29. If you have experience with VR, how many hours of VR use have you accumulated? (1-4)

30. Was femoral cannulation performed before the sternal incision? (y/n)

31. If yes, did you ask the perfusionist to empty the heart before dissecting the adhesions? (y/n)

32. Which surgical incision was chosen?

33. Did the preoperative VR planning influence the choice of surgical incision/approach? (y/n)

34. To what extent did the VR model help you choose the surgical approach/incision? (1-10)

35. Did the VR simulation change the operating room setup in any way? (y/n)

36. Did the VR simulation change your decision to use CPB? (y/n)

37. In your opinion, to what extent was the 3D model of the retrosternal anatomy accurate? (1-10)

38. Did the preoperative VR planning impact decision-making with respect to the operation? (y/n)

39. Did the VR model add value to the CT imaging? (1-10)

40. In your opinion did the VR preoperative planning shorten the duration of the resternotomy? (y/n)

41. How much shorter?

42. Were there any complications during the procedure? (y/n)

43. If yes, what kind of complications?

44. In your opinion, to what extent could the preoperative VR planning help you avoid complications? (1-10)

45. To what extent did the VR model help you to have a better understanding of the retrosternal anatomical orientation before/during surgery? (1-10)

46. Please rate your confidence in VR during the procedure: (1-10)

47. Was your confidence level higher, lower, or the same compared to surgery without preoperative VR simulation? (h/l/s)

48. Would you like to have access to a VR simulation prior to your next resternotomy? (1-10)

49. To what extent was the VR simulation useful prior to chest surgery in general? (1-10)

50. To what extent do you feel VR is an effective tool for preoperative planning in chest surgeries? (1-10)

51. To what extent do you feel that VR simulations can improve residential training programs in chest surgery? (1-10)

52. To what extent do you feel the VR simulation can improve residents' training for resternotomy?

CPB=cardiopulmonary bypass; CT=computed tomography; 3D=three dimensional; VR=virtual reality
